# Bird community shifts associated with saltwater exposure in coastal forests at the leading edge of rising sea level

**DOI:** 10.1371/journal.pone.0216540

**Published:** 2019-05-09

**Authors:** Paul J. Taillie, Christopher E. Moorman, Lindsey S. Smart, Krishna Pacifici

**Affiliations:** Forestry and Environmental Resources Department, North Carolina State University, Raleigh, North Carolina, United States of America; Weyerhaeuser Company, UNITED STATES

## Abstract

Rising sea levels dramatically alter the vegetation composition and structure of coastal ecosystems. However, the implications of these changes for coastal wildlife are poorly understood. We aimed to quantify responses of avian communities to forest change (i.e., ghost forests) in a low-lying coastal region highly vulnerable to rising sea level. We conducted point counts to sample avian communities at 156 forested points in eastern North Carolina, USA in 2013–2015. We modelled avian community composition using a multi-species hierarchical occupancy model and used metrics of vegetation structure derived from Light Detection and Ranging (LiDAR) data as covariates related to variation in bird responses. We used this model to predict occupancy for each bird species in 2001 (using an analogous 2001 LiDAR dataset) and 2014 and used the change in occupancy probability to estimate habitat losses and gains at 3 spatial extents: 1) the entire study area, 2) burned forests only, and 3) unburned, low-lying coastal forests only. Of the 56 bird species we investigated, we observed parameter estimates corresponding to a higher likelihood of occurring in ghost forest for 34 species, but only 9 of those had 95% posterior intervals that did not overlap 0, thus having strong support. Despite the high vulnerability of forests in the region to sea level rise, habitat losses and gains associated with rising sea level were small relative to those resulting from wildfire. Though the extent of habitat changes associated with the development of ghost forest was limited, these changes likely are more permanent and may compound over time as sea level rises at an increasing rate. As such, the proliferation of ghost forests from rising sea level has potential to become an important driver of forest bird habitat change in coastal regions.

## Introduction

As sea level rises, terrestrial, freshwater-dependent ecosystems become increasingly exposed to saltwater, which can dramatically alter vegetation composition and structure [1]. In the temperate zone, the gradient in saltwater exposure is broadly represented by a transition from salt-tolerant herbaceous marshes that may be inundated with saltwater daily, to upland forests that may be exposed to saltwater only on a decadal time scale [2,3]. Increasing saltwater exposure in upland forests typically results in a gradual shift in vegetation structure and composition towards that of a brackish or saline marsh [4,5]. Because germinating tree seeds and seedlings have a lower tolerance to salinity than mature trees, limited regeneration can result from even mild saltwater exposure events [1]. As the severity and duration of saltwater exposure increases, mature trees may die from osmotic stress, leaving large stands of dead trees known as “ghost forests” [4,6]. The opening of the canopy and the release from competition with regenerating trees allows for the proliferation of salt-tolerant shrubs and herbaceous plants [5]. These vegetation changes resulting from saltwater exposure likely will have important implications for biodiversity conservation in coastal regions.

Despite increasing concern over forest loss and the proliferation of ghost forests, few studies have investigated the value of ghost forests for birds and other wildlife. Many studies have investigated the implications of sea level rise for wildlife, but much of this work has focused on species associated with the immediate coastal environments such as marshes and mangroves [7]. Virtually all studies investigating the implications of sea level rise for birds in temperate regions have focused on beach-nesting birds [7–9], shorebirds [10,11], or marsh birds [7,12–16]. The landward migration of brackish vegetation communities may be essential to the persistence of these birds of the immediate coastal zone as sea level rises [7]. Moreover, active facilitation of landward migration even may be necessary if sea level rises faster than coastal communities can migrate [17]. However, understanding the tradeoffs for wildlife using the forests that migrating marshes would replace is requisite to developing sound conservation strategies. In addition to better understanding the potential benefits of facilitating marsh migration for birds associated with coastal marshes, these conservation strategies require consideration of the potential negative implications for forest birds that may be displaced as marshes migrate.

The well-established relationships between birds and vegetation [18–20] suggest the novel disturbance regimes related to rising sea level will have important implications for bird communities in coastal forests. The vegetation changes resulting from increased saltwater exposure in coastal forests may eliminate habitat for many birds, such as those associated with the upper layers of the forest canopy, but other species may benefit given disturbances such as wildfires and tropical cyclones are important ecological processes in coastal forests [21,22]. Many forest birds are specifically associated with the low canopy cover, standing dead wood, and early seral vegetation conditions following disturbance [23–25]. As such, saltwater exposure may potentially benefit disturbance-dependent birds by creating conditions resembling those created by other forms of disturbance, such as fire. Alternatively, ghost forests may differ from fire-created vegetation communities and provide novel conditions for a unique community of birds. Understanding how forest birds respond, whether positively or negatively, to ghost forests is requisite to weighing the conservation trade-offs of mitigating or facilitating the changes associated with novel disturbance regimes resulting from climate change.

To better understand the value of ghost forests as habitat for birds and the implications for coastal bird conservation, we conducted bird surveys across a gradient from severely saltwater-affected forests to unaffected forests in a region of North America that is extremely vulnerable to rising sea level. We used these observations to understand how bird communities varied across a gradient from forests most severely affected by saltwater exposure to those not affected. We modelled occupancy of forest birds using a hierarchical community model with metrics of vegetation structure measured by light detection and ranging (LiDAR) in 2014 and fire history as covariates on species-specific occupancy. Additionally, we aimed to use bird community modelling results to estimate the decadal-scale change in bird habitat associated with rising sea level. By spatially extrapolating the modelled relationships between vegetation structure and bird occupancy at the locations where we sampled birds to the broader extent of our study region, we estimated the occupancy of each bird species across the entire study area. Then, we used analogous LiDAR vegetation data from 2001 to predict bird occupancy at that time, allowing us to calculate the change in occupancy over a 13-year timeframe. Because habitat change across the study area likely has resulted from a variety of stressors (e.g., timber harvest, wildfire, hurricanes), we also estimated the change in occupancy within 2 more narrow spatial extents to focus on specific drivers of forest change: sea-level rise and fire. The goal of estimating this decadal-scale habitat change was to evaluate how these changes associated with sea level rise compared to broader responses across the region.

## Materials and methods

### Study area

The Albemarle-Pamlico Peninsula (APP) in eastern North Carolina, USA is bounded by the Albemarle Sound to the north, the Croatan Sound to the east, and the Pamlico Sound to the south. Along with the mouth of the Neuse River, these water bodies comprise the second-largest estuary complex in North America. The geomorphology of the estuary results in a gradual salinity gradient and limited influence of astronomic tides [2]. Instead, water movement is driven primarily by wind tides [26]. The limited number of inlets connecting the sounds to the ocean creates a gradient in salinity from the moderate salinity (10–18 ppt) Pamlico Sound to the largely freshwater (<3 ppt) Albemarle Sound [27]. Current rates of sea level rise in the estuary are ~4 mm/yr [28,29], up from ~2 mm/yr at the beginning of the 20^th^ century [30]. In addition to accelerating relative sea level rise, much of the APP lies below 1-m elevation, which means that small increases in sea level can have disproportionate effects on terrestrial environments. Furthermore, widespread ditches and canals installed to facilitate drainage often act as conduits for saltwater to move inland during saltwater intrusion events [26].

The forested wetlands of the APP varied from cypress (*Taxodium distichum*) and tupelo (*Nyssa* spp.) swamps in the bottomlands to loblolly pine (*Pinus taeda*) and pond pine (*Pinus serotina*) forests where soils were better drained [31]. Across the APP, soils are high in organic matter, with many areas having exclusively organic soils. Historically, fire return intervals ranged from 1–5 years in the herbaceous marshes and canebrakes to >100 years in bottomland forests. Fire return intervals in peatland pine forests ranged from 13–50 years [21]. In recent decades, fires included both wildfires and prescribed fires, but return intervals may be longer than historical [32]. In contrast to more xeric pine uplands in the southeastern United States, prescribed fires in pine-dominated forested wetlands often burn more intensely and create conditions more similar to wildfires [32,33].

### Site selection

**Fig 1:**
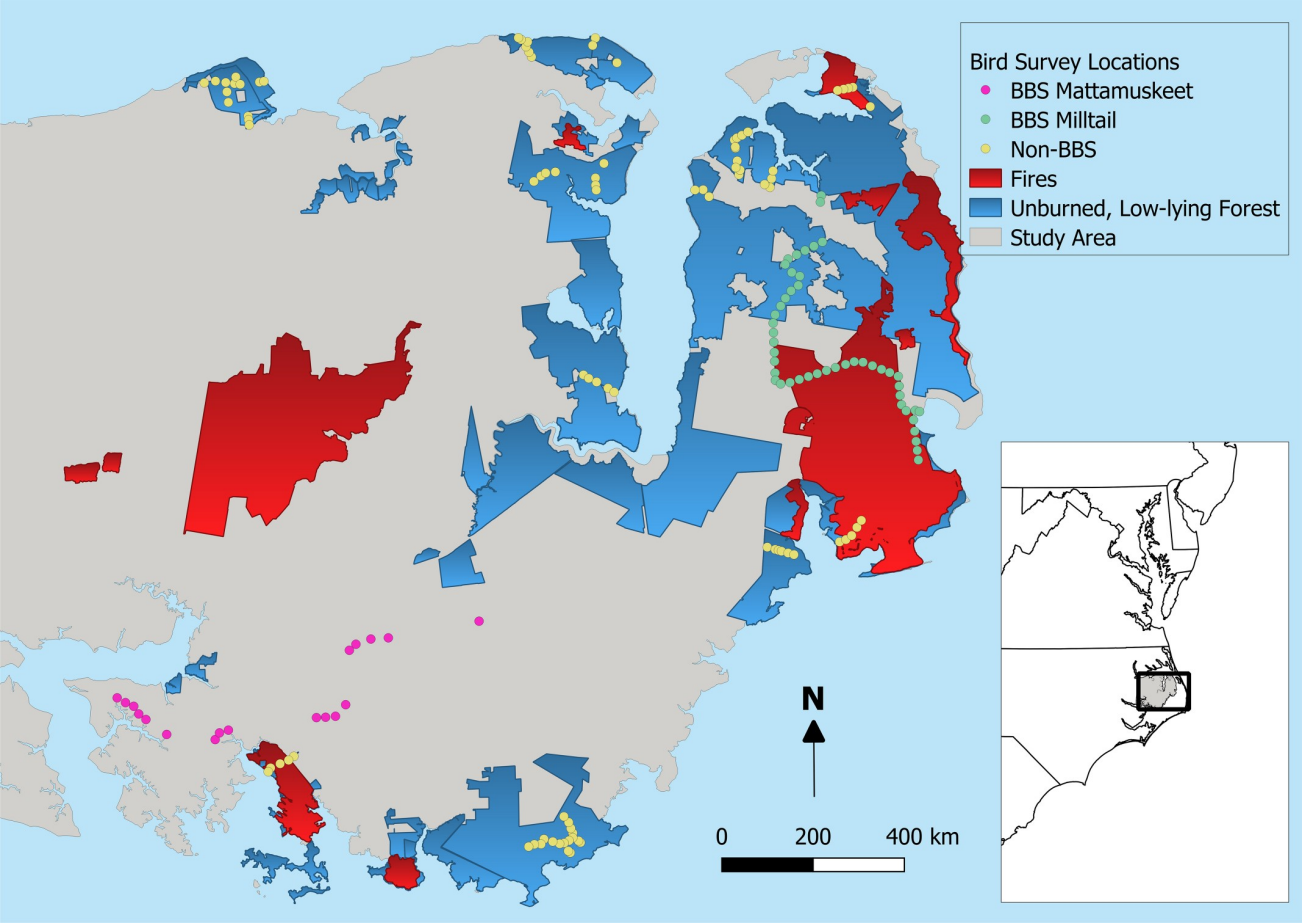
Bird survey sites on the Albemarle-Pamlico Peninsula in eastern North Carolina, USA shown according to BBS route, along with perimeters of all fires that burned between 2001 and 2014 (red) and the extent of publicly-owned forest within 1.5 m of sea level (blue).

We combined bird observations from the North American Breeding Bird Survey (BBS)[34] with additional monitoring sites selected specifically in parts of the APP most vulnerable to sea level rise. The BBS sites consisted of the 50 “stops” from each of the 2 BBS routes located on the outer APP. The Milltail Creek BBS route is largely along dirt roads within Alligator River National Wildlife Refuge and Dare County Bombing Range, though the last 7 stops are along Highway 264. The Lake Mattamuskeet BBS route is all on paved, low-traffic roads. We removed all stops from the 2 BBS routes that were comprised of >25% agriculture fields within 100 m (35 of 100 stops) to focus our study on primarily forested environments, leaving a total of 65 BBS sites (Fig 1). To supplement the BBS observations with bird monitoring sites in additional areas vulnerable to sea level rise, we selected additional points following selection protocols similar to the BBS, namely that sites were located along low-traffic roads without non-forest vegetation types within 100 m. We first identified all low-traffic, public roads on the APP within 1.5 m of sea level and that were undeveloped on both sides of the road. Because surveys were conducted from public roads, permission to access survey locations was not required. Nonetheless, we obtained research permits from the United States Fish & Wildlife Service, North Carolina Wildlife Resources Commission, and The Conservation Fund. We then randomly selected points along qualifying road segments with a minimum distance between points of 400 m, resulting in 91 non-BBS points, for a total of 156 points (Fig 1). Geographical coordinates of the survey locations can be found with the archived data online (Zenodo Online Data Repository doi:10.5281/zenodo.2548860)

### Bird surveys

At all 156 points, we recorded the number of every bird species detected during a 3-minute survey, following BBS protocol [34]. The 65 BBS points were surveyed once annually in 2013, 2014, and 2015. The non-BBS points were sampled 1 to 3 times in 2015. In all years, all surveys were conducted during the peak breeding season between 28 May and 19 June within 4 hours of sunrise. The observer for each of the BBS points was the same across the 3 years, but different between the 2 routes. A third observer conducted all of the bird surveys at the non-BBS points.

### Vegetation metrics

We used LiDAR data to quantify changes in vegetation structure across a gradient of saltwater exposure. We extracted 7 metrics of vegetation structure from LiDAR collected in 2014 for the North Carolina Floodplain Mapping Program to use as predictors of bird occupancy. Specifically, we calculated 3 height-based metrics (mean height, maximum height, and the standard deviation in height) and 4 metrics based on vegetation density within 1 of 4 height ranges (4.5–10 m, 10–20 m, 20–30 m, and total vegetation density). The density-based metrics were calculated by dividing the number of LiDAR returns in a 12-m by 12-m grid cell within a given height band by the total number of returns for that grid cell. The total vegetation density was calculated by dividing the number of all non-ground returns by the total number of returns for each 12-m by 12-m grid cell.

Because a preliminary analysis revealed a high degree of correlation among the vegetation metrics, we aimed to select a subset of the 7 metrics that best reflected the gradient from ghost forests to forests unaffected by saltwater exposure. To this end, we extracted values of the 7 metrics at randomly selected points within 1.5 m of sea level, outside of areas affected by urban development, timber harvest, agriculture, or fire since 2001, and spaced at least 400 m apart, resulting in a total of 248 points. We categorized all random points as either unaffected forest or ghost forest according to an increase or decrease, respectively, in the LiDAR-derived change in biomass from 2001 to 2014. We modelled this binary response of unaffected/ghost forest using logistic regression and employed a step-wise variable selection procedure to identify the vegetation metrics that best predicted this response. Because we observed collinearity among multiple variables, the standard practice of removing variables that were collinear was subjective and the final model depended strongly on which of the collinear variables were removed. Hence, the objective of this step-wise approach was to more objectively determine the best variables to include. In the first step, we fit a model for each of the 7 vegetation metrics and selected that with the lowest Akaike’s Information Criterion (AIC). We then fit additional models with the metric selected in step 1 and each of the other 6 metrics with a Pearson’s Correlation Coefficient less than 0.5, and again selected the best model according to lowest AIC. We repeated this procedure until no uncorrelated variables remained. If the addition of a variable did not decrease AIC, we assumed the additional variable was not informative and selected the more parsimonious model. We evaluated the predictive power of the final model by splitting the data into a training set (60%) and a validation set (40%) using the R package *caret* [35]. We used the training set to fit the model and predict whether or not each point in the validation set was ghost forest or not. We then compared the predictions to the observed state using two metrics of predictive ability for logistic regression models: sensitivity and specificity.

Rather than directly comparing bird communities in forests affected by saltwater with unaffected forests, we aimed to more explicitly relate bird community responses to the underlying vegetation conditions. Quantifying vegetation structure over a vast area in this way allowed us to make inferences about the relative contribution of the proliferation of ghost forests to broader, peninsula-wide patterns. Additionally, the availability of repeat LiDAR allowed us to investigate decadal scale change in avian habitat over time. Consideration of these large spatial and temporal scales would be impractical for a more direct comparison between ghost forest and unaffected forest [20].

### Bird community modelling

After identifying how vegetation structure varied between ghost forests and unaffected forests, our next step was to quantify how bird communities responded to these vegetation changes. To quantify bird community composition and investigate species-specific occupancy in terms of the vegetation structure metrics selected above, we used a Bayesian framework for modeling multi-species occupancy described by Royle and Dorazio [36]. Similar to other commonly used hierarchical occupancy models, this model assumes that the probability of detecting an individual (*p*) is not perfect (*p*<1). We used the detection history over the repeated visits at a given site to estimate the detection probability. Specifically, each of the *i* = 1,2,…*M* sites are visited *j* = 1,2,…*J* times, and the identities of *k* = 1,2…*K* species are recorded as detected during a sampling occasion. Because the BBS points only were sampled once annually, we pooled the 3 years of data into a single season with 3 sampling occasions, thus making the assumption that the occupancy state did not change over that time. We acknowledge that the occupancy state for a given species at a given point may change between years, but changes in occupancy from one year to the next are less relevant given our interest in the more gradual, decadal time-scale of the ecological changes resulting from sea level rise. Regardless, the BBS points comprised roughly 40% of bird sampling points, so we believe a violation of our assumption would have only a minimal effect on results. Following a similar approach to previous studies that investigated multi-species occupancy [37,38], we estimated the species-specific probability of occurrence at a given site (*ψ*_ik_), as well as species-specific covariate effects, by incorporating species-level parameters as random effects governed by community-level hyperparameters. Prior to model fitting, we omitted 35 of the 91 bird species detected because they were either non-native (e.g., European starling–*Sturnus vulgaris*), not associated with forest (e.g., white ibis–*Eudocimus albus*), or were otherwise not appropriately sampled with our protocol (e.g., flyovers such as chimney swift–*Chaetura pelagica*; Table 1). For the 56 remaining bird species, we converted all species-specific counts during a given survey to either detection (1) or non-detection (0).

**Table 1:** Common names, 4-letter codes, and scientific names of the 91 bird species detected on the Albemarle-Pamlico Peninsula in eastern North Carolina, USA (2013–2015).

Common name	Code	Genus	Species	Included^a^
Acadian Flycatcher	ACFL	*Empidonax*	*virescens*	Yes
American Crow	AMCR	*Corvus*	*brachyrhynchos*	No
American Goldfinch	AMGO	*Spinus*	*tristis*	Yes
American Robin	AMRO	*Turdus*	*migratorius*	Yes
Bald Eagle	BAEA	*Haliaeetus*	*leucocephalus*	No
Barn Swallow	BARS	*Hirundo*	*rustica*	No
Barred Owl	BARO	*Strix*	*varia*	No
Black-throated Green Warbler	BTNW	*Setophaga*	*virens*	Yes
Blue Grosbeak	BLGR	*Passerina*	*caerulea*	Yes
Blue Jay	BLJA	*Cyanocitta*	*cristata*	Yes
Blue-gray Gnatcatcher	BGGN	*Polioptila*	*caerulea*	Yes
Boat-tailed Grackle	BTGR	*Quiscalus*	*major*	Yes
Brown Thrasher	BRTH	*Toxostoma*	*rufum*	Yes
Brown-headed Cowbird	BHCO	*Molothrus*	*ater*	Yes
Brown-headed Nuthatch	BHNU	*Sitta*	*pusilla*	Yes
Canada Goose	CAGO	*Branta*	*canadensis*	No
Carolina Chickadee	CACH	*Poecile*	*carolinensis*	Yes
Carolina Wren	CARW	*Thryothorus*	*ludovicianus*	Yes
Cedar Waxwing	CEDW	*Bombycilla*	*cedrorum*	No
Chimney Swift	CHSW	*Chaetura*	*pelagica*	No
Chipping Sparrow	CHSP	*Spizella*	*passerina*	Yes
Chuck-will's-widow	CWWI	*Antrostomus*	*carolinensis*	No
Clapper Rail	CLRA	*Rallus*	*crepitans*	No
Common Grackle	COGR	*Quiscalus*	*quiscula*	Yes
Common Nighthawk	CONI	*Chordeiles*	*minor*	No
Common Yellowthroat	COYE	*Geothlypis*	*trichas*	Yes
Double-crested Cormorant	DCCO	*Phalacrocorax*	*auritus*	No
Downy Woodpecker	DOWO	*Picoides*	*pubescens*	Yes
Eastern Bluebird	EABL	*Sialia*	*sialis*	Yes
Eastern Kingbird	EAKI	*Tyrannus*	*tyrannus*	Yes
Eastern Meadowlark	EAME	*Sturnella*	*magna*	Yes
Eastern Towhee	EATO	*Pipilo*	*erythrophthal*	Yes
Eastern Wood-Pewee	EAWP	*Contopus*	*virens*	Yes
European Starling	EUST	*Sturnus*	*vulgaris*	No
Field Sparrow	FISP	*Spizella*	*pusilla*	Yes
Fish Crow	FICR	*Corvus*	*ossifragus*	No
Forster's Tern	FOTE	*Sterna*	*forsteri*	No
Glossy Ibis	GLIB	*Plegadis*	*falcinellus*	No
Gray Catbird	GRCA	*Dumetella*	*carolinensis*	Yes
Great Blue Heron	GBHE	*Ardea*	*herodias*	No
Great Crested Flycatcher	GCFL	*Myiarchus*	*crinitus*	Yes
Green Heron	GRHE	*Butorides*	*virescens*	No
Hairy Woodpecker	HAWO	*Picoides*	*villosus*	Yes
Hooded Warbler	HOWA	*Setophaga*	*citrina*	Yes
House Sparrow	HOSP	*Passer*	*domesticus*	No
House Wren	HOWR	*Troglodytes*	*aedon*	Yes
Indigo Bunting	INBU	*Passerina*	*cyanea*	Yes
Killdeer	KILL	*Charadrius*	*vociferus*	No
Laughing Gull	LAGU	*Leucophaeus*	*atricilla*	No
Least Bittern	LEBI	*Ixobrychus*	*exilis*	No
Little Blue Heron	LBHE	*Egretta*	*caerulea*	No
Mourning Dove	MODO	*Zenaida*	*macroura*	Yes
Northern Bobwhite	NOBO	*Colinus*	*virginianus*	Yes
Northern Cardinal	NOCA	*Cardinalis*	*cardinalis*	Yes
Northern Flicker	NOFL	*Colaptes*	*auratus*	Yes
Northern Mockingbird	NOMO	*Mimus*	*polyglottos*	Yes
Northern Parula	NOPA	*Setophaga*	*americana*	Yes
Northern Rough-winged Swallow	NRWS	*Stelgidopteryx*	*serripennis*	No
Orchard Oriole	OROR	*Icterus*	*spurius*	Yes
Osprey	OSPR	*Pandion*	*haliaetus*	No
Ovenbird	OVEN	*Seiurus*	*aurocapilla*	Yes
Pileated Woodpecker	PIWO	*Dryocopus*	*pileatus*	Yes
Pine Warbler	PIWA	*Setophaga*	*pinus*	Yes
Prairie Warbler	PRAW	*Setophaga*	*discolor*	Yes
Prothonotary Warbler	PROW	*Protonotaria*	*citrea*	Yes
Purple Martin	PUMA	*Progne*	*subis*	No
Red-bellied Woodpecker	RBWO	*Melanerpes*	*carolinus*	Yes
Red-cockaded Woodpecker	RCWO	*Picoides*	*borealis*	Yes
Red-eyed Vireo	REVI	*Vireo*	*olivaceus*	Yes
Red-headed Woodpecker	RHWO	*Melanerpes*	*erythrocephal*	Yes
Red-shouldered Hawk	RSHA	*Buteo*	*lineatus*	Yes
Red-tailed Hawk	RTHA	*Buteo*	*jamaicensis*	No
Red-winged Blackbird	RWBL	*Agelaius*	*phoeniceus*	Yes
Royal Tern	ROTE	*Thalasseus*	*maximus*	No
Ruby-throated Hummingbird	RTHU	*Archilochus*	*colubris*	Yes
Sharp-shinned Hawk	SSHA	*Accipiter*	*striatus*	No
Snowy Egret	SNEG	*Egretta*	*thula*	No
Summer Tanager	SUTA	*Piranga*	*rubra*	Yes
Swainson's Warbler	SWWA	*Limnothlypis*	*swainsonii*	Yes
Tricolored Heron	TRHE	*Egretta*	*tricolor*	No
Tufted Titmouse	TUTI	*Baeolophus*	*bicolor*	Yes
Turkey Vulture	TUVU	*Cathartes*	*aura*	No
White Ibis	WHIB	*Eudocimus*	*albus*	No
White-eyed Vireo	WEVI	*Vireo*	*griseus*	Yes
Wild Turkey	WITU	*Meleagris*	*gallopavo*	No
Willet	WILL	*Tringa*	*semipalmata*	No
Wood Duck	WODO	*Aix*	*sponsa*	No
Worm-eating Warbler	WEWA	*Helmitheros*	*vermivorum*	Yes
Yellow-billed Cuckoo	YBCU	*Coccyzus*	*americanus*	Yes
Yellow-breasted Chat	YBCH	*Icteria*	*virens*	Yes
Yellow-throated Warbler	YTWA	*Setophaga*	*dominica*	Yes

^a^ whether or not a species was included in analyses of bird community composition.

We investigated variation in occupancy and detection by incorporating covariates on these processes as linear combinations of the logit transform of occupancy probability and detection probability, respectively. Because each bird survey route was surveyed by a unique observer and may have varied according to other factors affecting detection (e.g., traffic volume), we included a categorical covariate on detection probability corresponding to either one of the BBS routes or the non-BBS sites (Eq 1). We included the vegetation metrics selected in the stepwise selection procedure above as covariates on each species’ occupancy probability. We accounted for the effect of fire (both wildfire and prescribed fire) by including a binary covariate of whether or not the site burned between 2001 and 2014 (Eq 2). We standardized all continuous variables prior to model fitting by subtracting the mean and dividing by the standard deviation. With the mean of standardized variables equal to 0, the inverse-logit of the species-specific intercepts (*β*_*0k*_) represents the occupancy probability for each species at the average value of those covariates.

$$logit(p_{ijk})=\alpha_{0k}+\alpha_{1k}*{Route}_{i}$$ 1)

$$logit(\psi_{ik})=\beta_{0k}+\beta_{1k}*{LiDAR\;Metric1}_{i}+\beta_{2k}*{LiDAR\;Metric2}_{i}+\beta_{3k}*Burned_{i}$$ 2)

We fit the community model using the R package *rjags* [39,40]. We used vague priors (normal distribution with mean = 0 and variance = 1000) for all community-level hyperparameters. We ran 3 MCMC chains for 20,000 iterations after an initial burn-in of 5000 iterations. We thinned the MCMC chains to every 5^th^ iteration to account for autocorrelation between adjacent MCMC samples. We evaluated model convergence by investigating trace plots and the Gelman-Rubin diagnostic for all parameters [40]. To distinguish the species-specific covariate effects with the most support, we considered 95% posterior distribution credible intervals that included 0 to be weakly supported.

In addition to investigating species-specific responses, we evaluated the conservation value of different bird communities according to the modeled species richness and a metric of conservation priority averaged across the species in that community. Though we only investigated a subset of species that we detected, this metric serves as an informative measure of species richness nonetheless. Within each MCMC iteration, we summed the site-specific occupancy state (*z*_*ik*_) across the 56 species to get a posterior distribution of species richness at each site. Additionally, we were interested in how species richness was related to each of the vegetation metrics that we used to describe differences between unaffected forests and ghost forests. We assessed the relationship between species richness and each vegetation metric using linear regression by fitting a separate linear model for each metric with species richness as the dependent variable. We compared the conservation priority of birds responding positively to ghost forest to those that responded negatively to ghost forest using the Partners-in-flight (PIF) Conservation Priority Score, an expert-derived metric based on several categories of demographics for each individual species [41]. The PIF score has proven to be a useful indicator for focusing management attention on the species most in need of conservation action[41]. Using the species-specific responses to the metrics of vegetation structure that we estimated, we grouped bird species into those with either strong positive or strong negative support for an association with ghost forest. Within these groups, we then calculated the average PIF score to quantify the relative conservation value of the species groups.

### Habitat loss/gain

We predicted occupancy probability for each bird species across the extent of the APP using the species-specific parameter estimates of covariate effects and the peninsula-wide values of the LiDAR metrics. We repeated this procedure using analogous LiDAR-derived vegetation metrics calculated in 2001, allowing us to then estimate the change in occupancy probability from 2001 to 2014 (*Δψ*). However, we assumed that the bird species responses to vegetation conditions did not change over this time, and also that the bird communities in 2001 did not have additional species that we did not detect in 2013–2015. In order to speed computation time for these peninsula-wide predictions, we first reduced the resolution of the LiDAR metric rasters to 48 m by 48 m. To account for uncertainty in the estimated change in occupancy, we categorized changes in occupancy probability based on a threshold of 0.3, such that the sum of the grid cells that decreased in occupancy probability by at least 0.3 represented the total habitat loss for a given species (Eq 3), and the sum of those that increased by at least 0.3 represented habitat gain (Eq 4). Rather than simply considering species whose occupancy increased or decreased, use of the 0.3 threshold results in more conservative estimates of habitat losses and gains.

In addition to calculating the total peninsula-wide habitat loss and gain for each species, we calculated habitat loss and gain at 2 additional spatial extents–within wildfires, and within unburned forests vulnerable to saltwater. To estimate habitat loss and gain attributable to fire, we calculated these values within the perimeter of any fire that burned between 2001 and 2014. To estimate habitat loss and gain in unburned forests that were most vulnerable to saltwater exposure, we calculated these values for forests within 1.5 m of sea level and that were not burned or harvested between 2001 and 2014. We considered only publicly owned properties and consulted with the associated land managers to identify and exclude the few areas on these public lands that were harvested during this time.

habitatloss=(∑cellswhereΔψ<−0.3)*0.2304ha 3)

habitatgain=(∑cellswhereΔψ>0.3)*0.2304ha 4)

## Results

The vegetation density between 4.5 and 10 m (hereafter: midstory density) was the single best predictor of ghost forest. With midstory density held fixed in all models (i.e., the second step of the stepwise model selection), the best additional predictor was vegetation density between 20 and 30 m (hereafter: canopy density), though the model with midstory density and mean height was within 2 AIC (Table 2). Vegetation density 10–20 m (i.e., lower canopy density) was not correlated with either of the 2 previously selected variables, but addition of this variable did not increase predictive power, reflected by a greater AIC (Table 2). Therefore the top-ranked model used for successive analysis included both midstory and canopy density. The sensitivity and specificity calculated for this model were 0.86 and 0.79, respectively. The complete set of covariates we used as predictors of bird occupancy were midstory density, canopy density, and a binary categorical variable of whether or not the site burned between 2001 and 2014. A site was more likely to have decreased in biomass from 2001 to 2014, thus representing ghost forest, if the 2014 vegetation density was lower in both the midstory (4.5 to 10 m) and canopy (20 to 30 m) layers.

**Table 2:** Stepwise logistic regression model selection procedure to determine the variables that best predicted ghost forest in eastern North Carolina (2014).

Step	Model	AIC	ΔAIC^a^
1			
	mean height	195.0	12.8
	max height	230.1	47.9
	sd height	211.1	28.9
	midstory density^b^	182.2	0
	lower canopy density^c^	248.4	66.1
	canopy density^d^	277.8	95.6
	total veg density	224.8	42.6
2			
	midstory density + mean height	164.2	0.47
	midstory density + max height	173.8	10.1
	midstory density + sd height	166.5	2.8
	midstory density + lower canopy density	178.1	14.4
	midstory density + canopy density	163.7	0
3			
	midstory density + canopy density + lower canopy density	165.7	-

^a^ Delta AIC is the difference between AIC values for the given model and the top-ranked model within the step.

^b^ Vegetation Density 4.5–10 m above ground.

^c^ Vegetation Density 10–20 m above ground.

^d^ Vegetation Density 20–30 m above ground.

**Fig 2:**
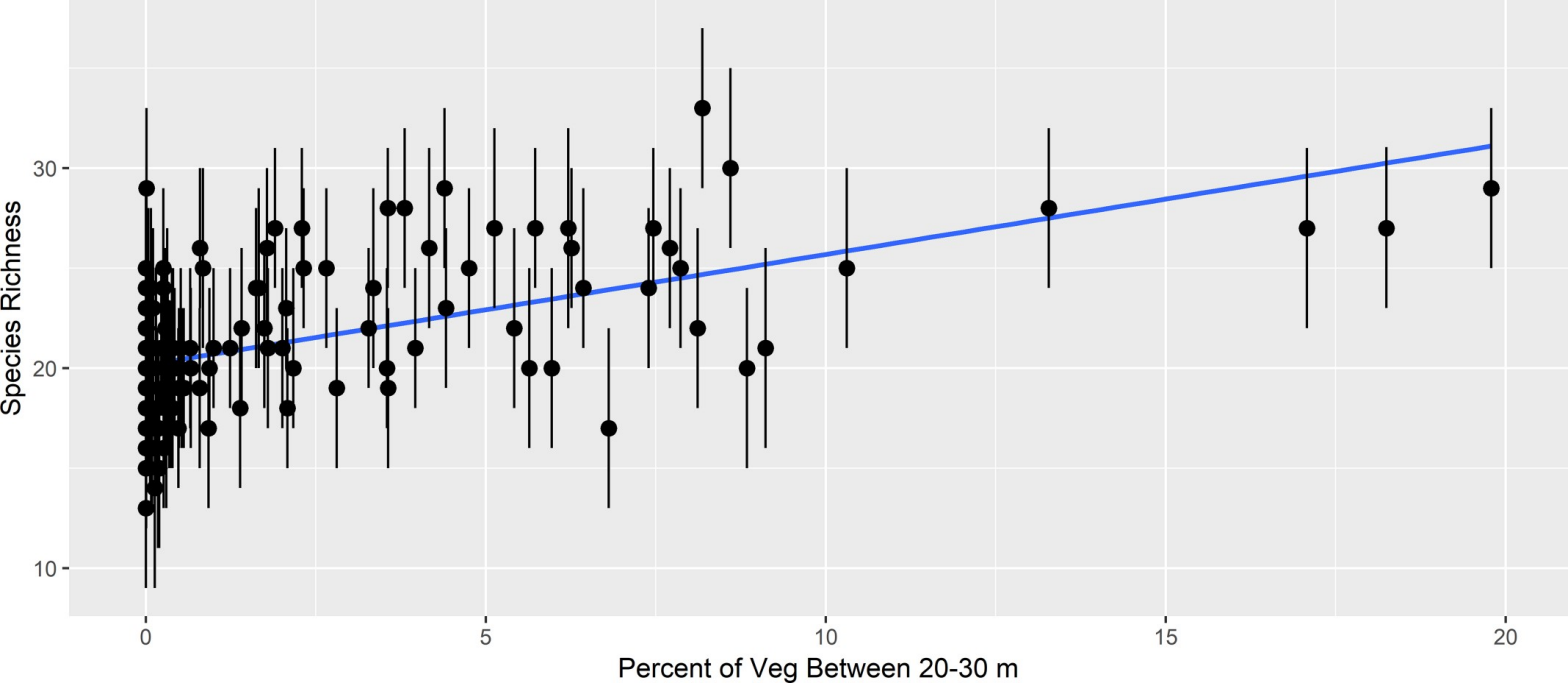
Posterior median species richness (estimated number of species at a given site of the 56 species we analyzed), shown with 95% credible intervals (error bars) and linear relationship (blue line), as a function of canopy density (vegetation from 20 to 30 m) at 156 points in coastal forests of eastern North Carolina, USA (2013–2015).

**Fig 3:**
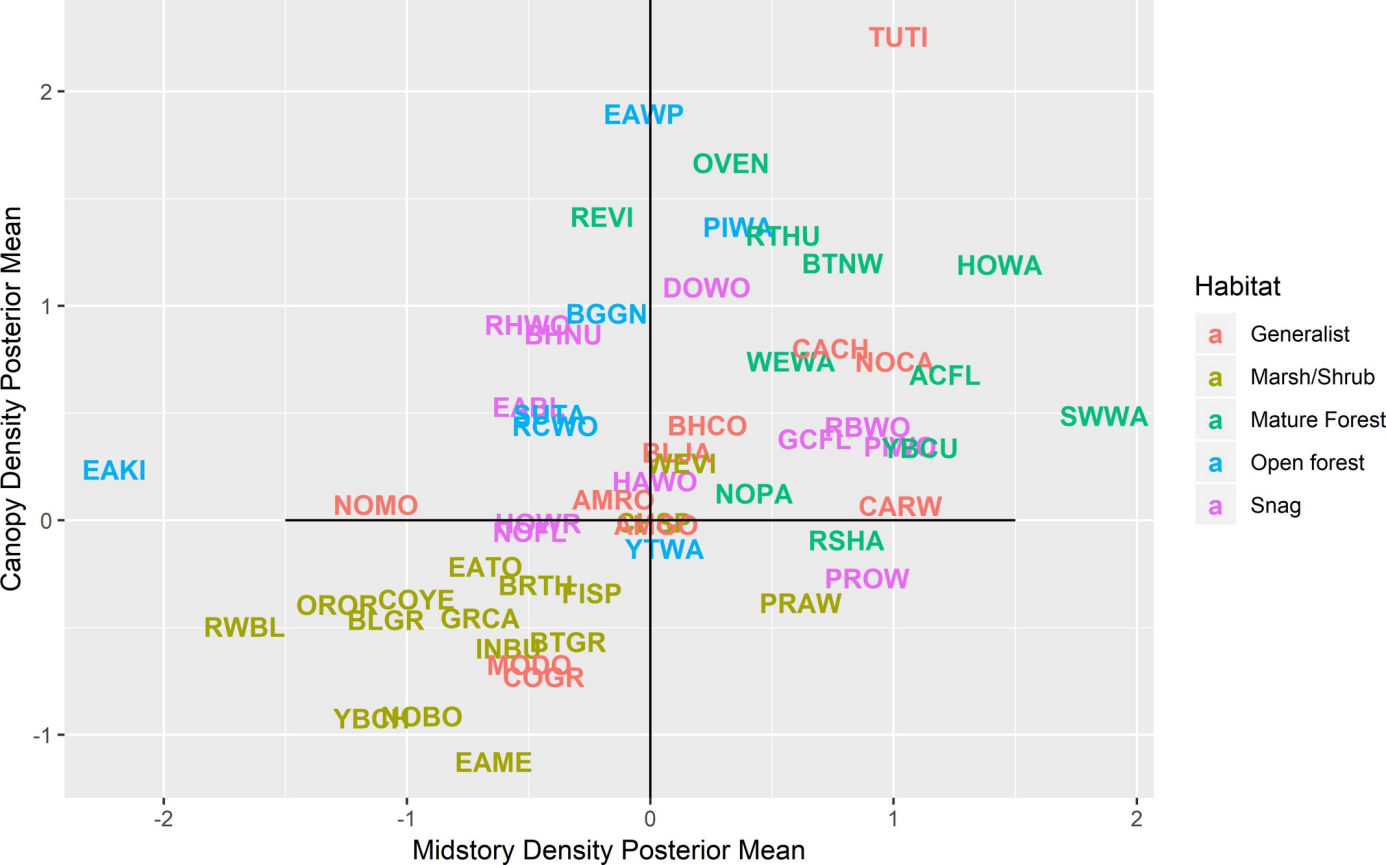
Posterior means for the effects of forest canopy and midstory vegetation density on occupancy of 56 species of birds detected in coastal forests of eastern North Carolina, USA between 2013 and 2015. The farther above/below or left/right of the zero lines (solid lines), represent a stronger response to canopy density and midstory density, respectively. Codes for bird species are located according to the magnitude and direction of the corresponding posterior means and are colored according to known habitat associations (generalist = multiple habitat associations, marsh/shrub = low statured vegetation, mature forest = closed canopy forest, open forest = open canopy forest, snag = standing dead trees). Common and scientific names of birds are in Table 1.

The estimated bird species richness (out of the possible 56 species we investigated) ranged from 13 to 33 across all points. We did not observe a relationship between estimated richness and midstory density (p value = 0.85), but richness increased with increasing canopy density (p value < 0.001; Fig 2). Because ghost forest was best predicted by less dense vegetation in both the canopy and midstory layers, species with negative parameter estimates for both of these occupancy covariates, such as red-winged blackbird, yellow-breasted chat, and common yellowthroat, were more likely to occur in ghost forests (Fig 3; scientific names of birds are in Table 1). Species such as Acadian flycatcher, black-throated green warbler, and hooded warbler had positive parameter estimates for both vegetation covariates, and thus were less likely to occur in ghost forests (Fig 3). Several species had parameter estimates for these covariates that were opposite in sign. For example, prothonotary warbler and prairie warbler were more likely to occur at sites with less vegetation density in the canopy, but greater density in the midstory (Fig 3). In contrast, brown-headed nuthatch, eastern bluebird, and red-headed woodpecker were more likely to occur at sites with greater canopy densities and less midstory densities (Fig 3). The average PIF conservation priority score for the species with at least one strongly supported positive effect of vegetation density was 9.2 (i.e., birds that occurred in unaffected forest), and the average for the species with at least one strongly supported negative effect was 9.1 (i.e., birds that occurred in ghost forest).

We observed support for a relationship (positive or negative) between bird occupancy and at least 1 of the 2 vegetation density covariates for 25 of the 56 bird species, but both covariates had support for only 2 species, northern bobwhite (both posterior means negative) and tufted titmouse (both posterior means were positive). There was support for an effect of midstory density, either positively or negatively, for almost twice as many species as the effect of canopy density (Table 3). Occupancy probability increased at greater vegetation densities in the midstory and canopy for 11 and 6 species, respectively (Table 3). In contrast, occupancy probability increased at lower vegetation densities in these layers for 7 and 3 species, respectively (Table 3). Thus, when considering only those 95% posterior credible intervals that did not overlap 0 for either vegetation metric, a greater number of species were more likely to occur where vegetation conditions reflected unaffected forests (16) than in ghost forest (9; Table 3).

**Table 3:** The number of bird species whose posterior means were less than 0 (more likely to occur in saltwater affected forest) and greater than 0 (more likely to occur in unaffected forest) for each of 2 vegetation density covariates on occupancy in eastern North Carolina, USA (2013–2015).

	Posterior mean <0	Posterior mean >0
Vegetation density 4.5–10 m	28 (7)^a^	28 (11)
Vegetation density 20–30 m	23 (3)	33 (6)
Either covariate	34 (9)	39 (16)
Both covariates	17 (1)	22 (1)

^a^ The number of species whose 95% credible intervals did not overlap 0 are shown in parentheses.

**Fig 4:**
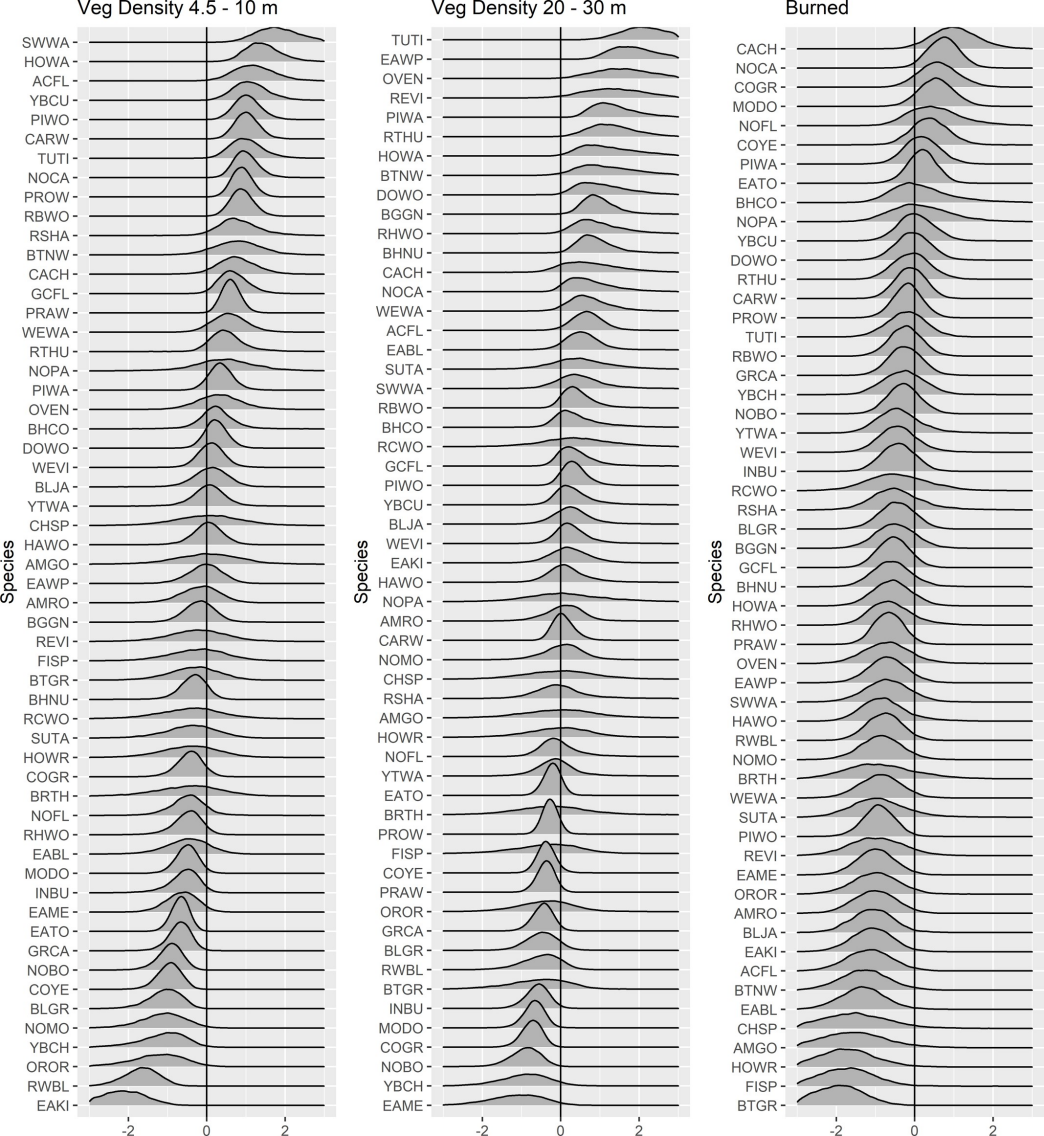
Posterior distributions of species-specific parameter estimates for each of 3 covariates (midstory density, canopy density, and burned/unburned) on occupancy probability for 56 species of forest birds detected in coastal forests in eastern North Carolina between 2013 and 2015.

The effect of fire did not explain much additional variation in occupancy for most species. The posterior distribution of the categorical burned forest covariate did not overlap 0 for only 5 of the 56 species, including boat-tailed grackle, field sparrow, chipping sparrow, house wren, and eastern bluebird, all of which were more likely to occur in unburned forests (Fig 4). Posterior means for this covariate were negative (i.e. more likely to occur in unburned forest) for most of the 56 species (78%). Carolina chickadee, northern cardinal, common grackle, and mourning dove had the most positive effects of burned forest, but each of these posterior credible intervals overlapped 0 (Fig 4).

**Fig 5:**
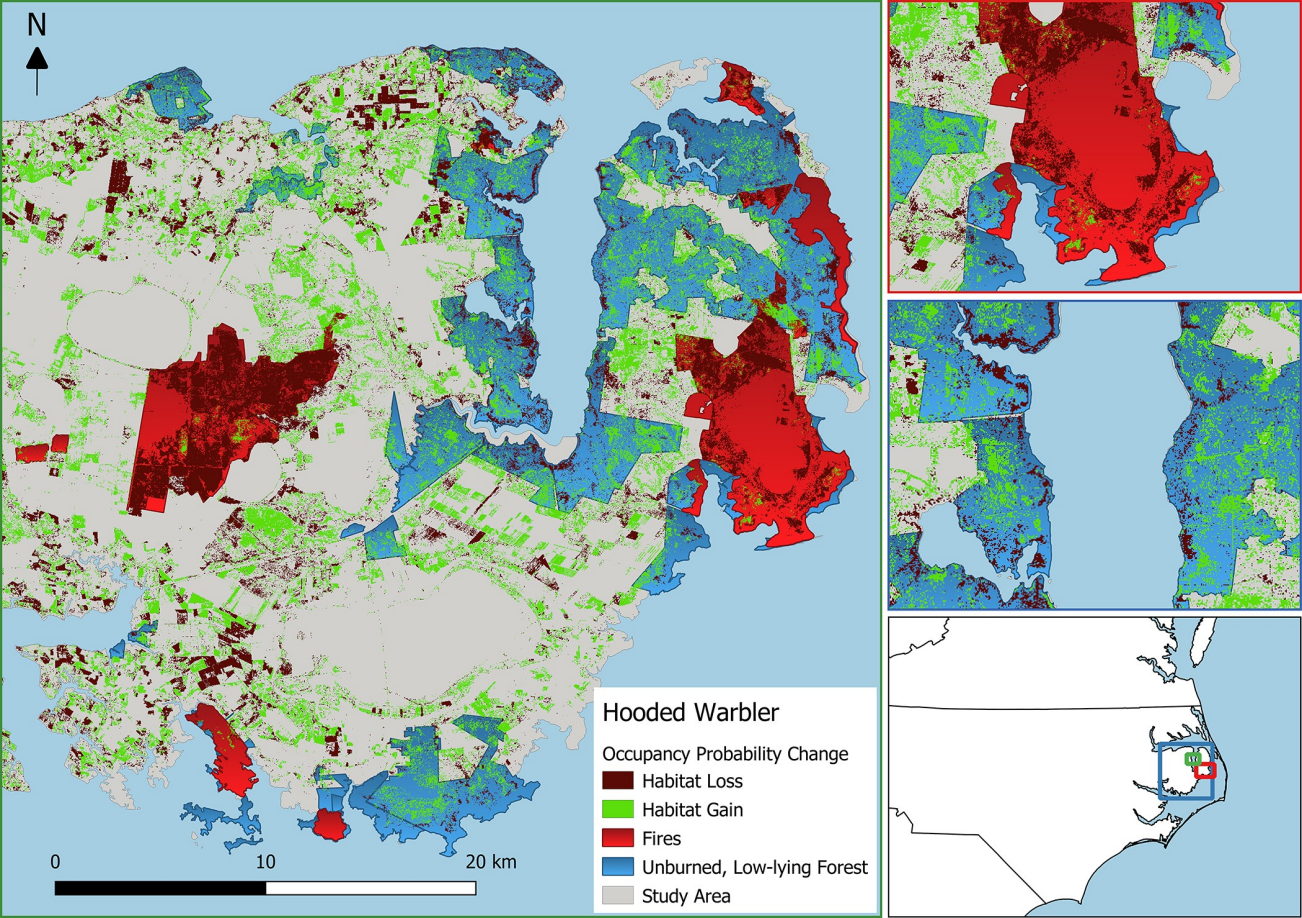
Estimated habitat losses (blue) and gains (green) for hooded warbler on the Albemarle-Pamlico Peninsula in eastern North Carolina, USA resulting from the LiDAR-derived changes in canopy (vegetation 20–30 m tall) and midstory (vegetation 4.5–10 m tall) density between 2001 and 2014. Changes in occupancy probability (modeled using a multi-species, hierarchical occupancy model) less than -0.3 were assumed to equate to habitat loss, whereas increases greater than 0.3 were assumed to equate to habitat gain. Prescribed fires and wildfires that burned between 2001 and 2014 shown in red and unburned, publicly owned forests within 1.5 m of sea level are shown in brown. Inset maps show the location of the study area (left), a smaller scale view of the Pain’s Bay wildfire that burned in 2011 (middle), and a smaller scale view of a portion of the Alligator River State Game Land.

**Fig 6:**
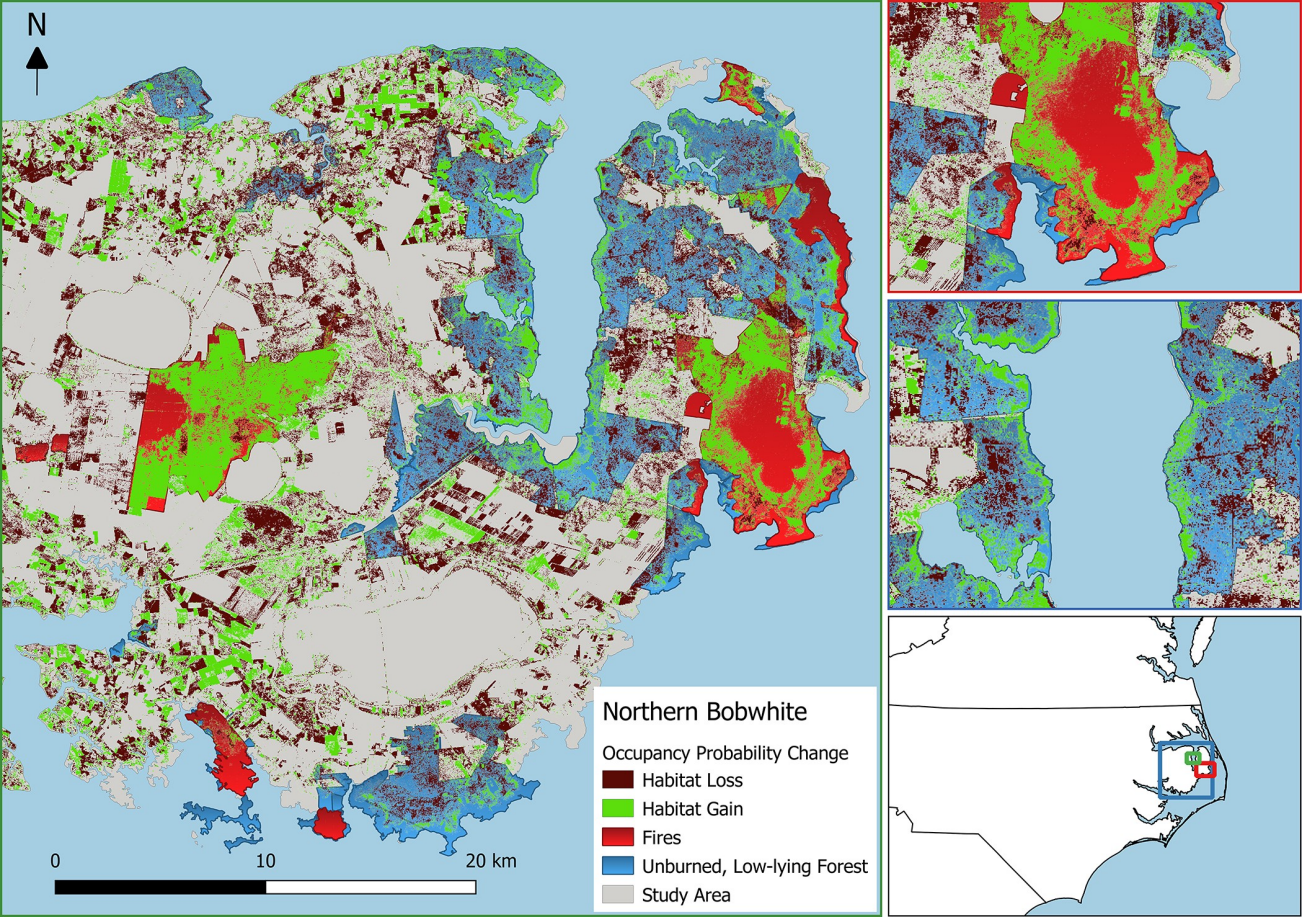
Estimated habitat losses (blue) and gains (green) for northern bobwhite on the Albemarle-Pamlico Peninsula in eastern North Carolina, USA resulting from the LiDAR-derived changes in canopy (vegetation 20–30 m tall) and midstory (vegetation 4.5–10 m tall) density between 2001 and 2014. Changes in occupancy probability (modeled using a multi-species, hierarchical occupancy model) less than -0.3 were assumed to equate to habitat loss, whereas increases greater than 0.3 were assumed to equate to habitat gain. Prescribed fires and wildfires that burned between 2001 and 2014 shown in red and unburned, publicly owned forests within 1.5 m of sea level are shown in brown. Inset maps show the location of the study area (left), a smaller scale view of the Pain’s Bay wildfire that burned in 2011 (middle), and a smaller scale view of a portion of the Alligator River State Game Land.

The amount of habitat lost or gained in unburned, low-lying forest was generally small compared to the overall change in habitat amount across the entire APP from 2001 to 2014 for most bird species (Table 4). The 3 species that experienced the most peninsula-wide habitat loss, eastern kingbird, red-winged blackbird, and blue grosbeak, were all species that were more likely to occur in ghost forest according to at least one of the vegetation density metrics (Table 4). In contrast, the 3 species that experienced the most habitat gains across the entire APP were more likely to occur in unaffected forests where vegetation densities in the canopy and midstory were greater. Furthermore, we observed contrasting trends in net habitat change (area gained—area lost) in forests that were burned when compared with unburned, low-lying forests. The 5 species with the largest net habitat gains in unburned, low-lying forests–Swainson’s warbler, hooded warbler, Acadian flycatcher, yellow-billed cuckoo, and northern cardinal–were the same 5 species with the largest net losses in burned areas (Table 4). In addition, these species with the largest habitat gains in low-lying coastal forests were those predicted to lose habitat from the proliferation of ghost forest based on responses to canopy and midstory vegetation density. Similarly, the 5 species with the largest net losses in unburned, low-lying forests–eastern kingbird, blue grosbeak, red-winged blackbird, common yellowthroat, and northern bobwhite–were more likely to occur in ghost forests; these same species gained the most habitat in burned forests (Table 4). Within unburned, low-lying forests, habitat gains for early seral species and losses for dense forest species were mostly limited to a narrow band at the interface between forest and marsh near the shoreline (Figs 5 and 6). In contrast, habitat change for these species resulting from fire was relatively consistent throughout burned areas (Figs 5 and 6).

**Table 4:** The estimated hectares of net habitat change from 2001 to 2014 for the 25 bird species whose occupancy was significantly predicted by at least one of the vegetation metrics related to ghost forest.

Species^a^	Total Change (ha)	Burned (ha)^b^	Low-lying Coastal Forest (ha)^c^	PIF Score^d^
ACFL	11,534	-13,863	6,009	11
BGGN	-8,536	-145	-2,052	7
BLGR	-12,549	15,300	-4,925	8
CARW	7,418	-5,877	314	7
COGR	2,883	7,119	-556	9
COYE	-8,062	12,354	-4,023	9
EAKI	-30,041	16,753	-10,385	11
EATO	-3,583	10,538	-819	11
EAWP	-6,945	-160	-1,773	10
GRCA	-2,935	11,421	-2,251	8
HOWA	11,096	-14,581	6,222	9
MODO	594	9,377	-1,062	6
NOBO	-7,245	14,513	-3,403	12
NOCA	8,462	-14,692	4,073	5
OVEN	-4,031	-3,897	170	9
PIWA	-5,878	-3,536	-283	7
PIWO	4,685	-10,387	245	7
PRAW	12,350	118	2,881	13
PROW	16,694	-2,477	3,181	14
RBWO	3,466	-9,647	215	7
RTHU	-1,070	-9,177	1,062	8
RWBL	-15,556	16,456	-4,906	8
SWWA	27,014	-15,954	10,956	13
TUTI	3,639	-12,835	3,286	7
YBCU	13,914	-15,360	4,570	12
Total	456,112	43,6909	137,055	

^a^ Species codes given in Table 1.

^b^ Habitat change in areas affected by fire between 2001 and 2014.

^c^ Habitat change in publicly owned forests within 1.5 m of sea level not affected by fire or timber harvest between 2001 and 2014.

^d^ Partners-in-flight (PIF) conservation priority scores.

## Discussion

Though ghost forests support a different avian community than the forests they replace, the habitat losses and gains associated with rising sea level appear to be small relative to the changes across the entire area we investigated, even though the region is highly vulnerable to rising sea level. Granted, birds more likely to occur in unaffected forests may continue to lose habitat as the extent of ghost forests increases, but the current rate of habitat loss for these species related to sea level rise is small compared to other drivers of vegetation dynamics (e.g., wildfire, fire suppression, forest maturation, and timber harvest). Thus, addressing other factors responsible for the declines of birds breeding in coastal forests, including urbanization, non-native plant invasions, and altered fire regimes, may be more effective strategies for conserving coastal forest birds in the near-term [42–44].

The birds most negatively affected by the development of ghost forest conditions were associated with multiple structural components of closed-canopy forests. Vegetation in the upper canopy provides foraging opportunities for canopy-dwelling species, such as black-throated green warbler, eastern wood-pewee, and pine warbler, whereas midstory vegetation has been shown to be an important structural component for Acadian flycatcher and hooded warbler [45–48]. Furthermore, increased vegetation density in these layers limits the amount of light reaching the forest floor, in turn limiting the development of understory vegetation. In a closed-canopy forest, the lack of understory and accumulation of deep litter can be beneficial for ground-foraging species, such as Swainson’s warbler [49]. The continued development of ghost forests will likely translate to increasing habitat losses for these birds associated with the structural components of closed-canopy forests.

As the moniker “ghost forest” implies, snags are an important component of these transitional vegetation communities. Thus, cavity-nesting and bark-foraging birds would be expected to be more common in snag-dense forests [50], yet the parameter estimates for the effects of canopy and midstory density on occupancy was near 0 for most of these birds. However, the vegetation density responses for snag-associated species were consistently intermediate between those of birds associated with early seral conditions and birds associated with more mature, unaffected forests. Thus, the 2 vegetation density covariates we considered likely reflect a broader transition of vegetation conditions from unaffected forests to shrubland and marsh, with ghost forests representing an intermediate stage along this transition, thus placing snag-associated species near the middle of this transition. An additional explanation for weak responses among snag-associated species could be that the variables we considered do not accurately reflect the most important habitat characteristics for these species (e.g., snag density). Further study investigating the more nuanced variation of vegetation characteristics in ghost forests, such as snag densities, could reveal stronger relationships between occupancy of cavity-nesting birds and ghost forest conditions [50].

Though sea level rise often is considered a threat to biodiversity in coastal regions, the proliferation of ghost forests appears to benefit a number of bird species. Furthermore, the conservation value of these birds that use ghost forests were comparable to that of birds in unaffected forests. Many of the birds we observed that were most likely to occur in ghost forests, including common yellowthroat, northern bobwhite, and yellow-breasted chat, are early seral species that rely on dense shrub and herbaceous cover [51–53]. The reduced canopy and midstory cover in ghost forests allows light to reach the understory, facilitating growth of grasses, forbs, and shrubs that are more tolerant than trees to saltwater exposure. Despite the creation of these conditions in ghost forests, we observed net habitat losses for many early seral birds, even in the most low-lying coastal forests. This net loss likely demonstrates the limited spatial scale of current ghost forests relative to the total amount of unburned, low-lying forest on the APP peninsula. Additionally, grass-forb-shrub communities may develop primarily at the later stages of the transition from forest to marsh, and given the relatively recent development of ghost forests, the extent of plant communities at these later transitional stages may be limited. Finally, peninsula-wide habitat loss for birds associated with forest disturbance may reflect a broader pattern of decline for these species, and small habitat gains associated with the proliferation of ghost forest may be masked by broader trends of forest maturation or fire suppression [23].

Our results help to contextualize the emerging role of saltwater exposure and sea level rise as agents of disturbance in coastal forests. Though the habitat losses and gains that we observed in unburned, low-lying forest were substantial for some species, they were small compared to those following severe wildfires in the region. Despite a low prevalence of prescribed fire and wildfire in our study area compared to historical estimates [21], fire appears to remain one of the most important drivers of bird habitat dynamics on the APP. Though the vegetation conditions caused by saltwater exposure and severe wildfire may be similar (e.g., high snag densities and dense understory cover), the successional trajectories of these 2 post-disturbance communities likely are different. Tree species that evolved in the presence of fire often have adaptations that allow them to regenerate rapidly post-fire, and as such, the vegetation conditions following wildfire can be short-lived [25,54]. Conversely, regeneration may be limited by low salinity tolerance of seedlings in ghost forests resulting from saltwater exposure [1]. Though saltwater exposure may occur in acute events, these events are likely to increase in frequency and severity as sea level rises [55]. Thus, ghost forests may continue on a trajectory towards conversion to salt-tolerant herbaceous marsh rather than return to forest. Though the decadal-scale changes in habitat extent from saltwater exposure may not be as extensive as those caused by fire, they likely are more permanent and may compound over future decades. Furthermore, as the extent of saltwater exposure moves further landward, the opportunity for the interaction between fire and saltwater exposure increases, which may speed the transition to herbaceous marsh and the associated loss of coastal forest [5].

Though the proportion of the avian community observed to respond positively or negatively to the vegetation characteristics associated with ghost forest was small, many additional species had moderate support for a relationship to these variables despite not meeting our established threshold. One of the advantages of hierarchical community models is the ability to make inferences about rare species; however, the responses of these rare species may tend towards the community mean [38], which in our case was near 0. As such, more focused surveys of the avian community, for example away from roads with multiple visits per year at a greater number of points, could potentially reduce uncertainty and reveal stronger support for responses by less-common species (e.g., worm-eating warbler and black-throated green warbler). An alternative explanation for the small proportion of strong responses could be that habitat characteristics we did not consider may be more appropriate predictors of occupancy for some species. In addition to a more direct quantification of snag density, the density of understory vegetation is an important component of ghost forests and has been shown to drive habitat use for several bird species in low-lying forests [52,56,57]. However, the accuracy of near-ground LiDAR returns often is unreliable, particularly for low return-density data such as that collected in 2001 [58]. Advances in remote sensing technology and incorporation of additional data, such as spectral imagery, may help to provide better information on the spatial variability in vegetation structure of coastal regions.

The rate of future sea level rise and the implications for coastal areas are inherently uncertain, which complicates the development of effective conservation strategies. Our study addresses this need by identifying the tradeoffs for coastal birds associated with land management strategies that may facilitate or mitigate the effects of saltwater exposure and sea level rise in coastal forests. Consideration of these tradeoffs likely will become increasingly important as rates of sea level rise increase.
